# Supplementary material for: A visual opsin from jellyfish enables precise temporal control of G protein signalling
Source: Nat Commun. 2023 Apr 28;14:2450. doi: 10.1038/s41467-023-38231-z (PMC10147646; doi:10.1038/s41467-023-38231-z)
Supplement: Supplementary file 1 — Supplementary Information [file 41467_2023_38231_MOESM1_ESM.pdf]

Supplementary Information for

A visual opsin from jellyfish enables precise temporal control of G protein signalling

Michiel van Wyk and Sonja Kleinlogel

Correspondence to: michiel.vanwyk@unibe.ch or sonja.kleinlogel@roche.com

Included:

Supplementary Figures 1 to 9

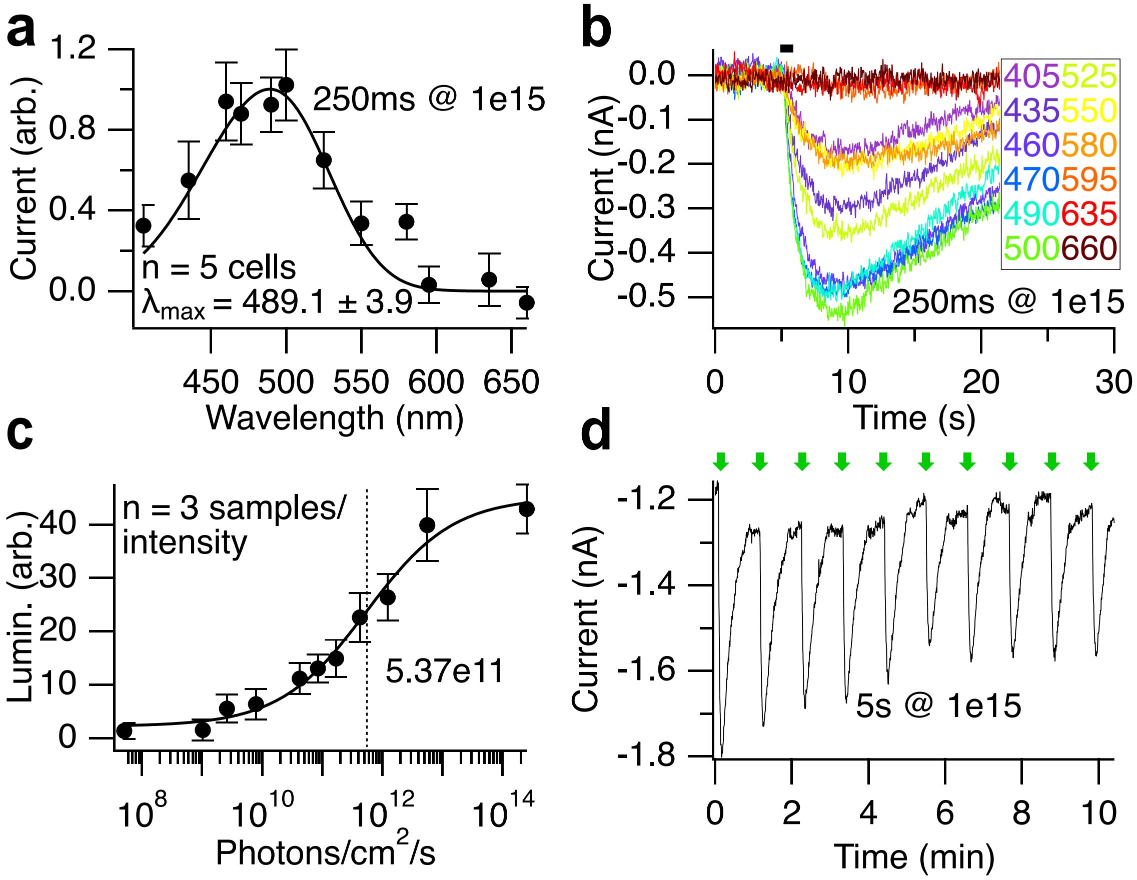

Supplementary Figure 1. **Light response properties of JellyOp. a** An action spectrum generated from GIRK response amplitudes with a Govardovskii fit predicts a maximum absorption of 489nm (show is mean ± SD). **b** Example traces from one of the HEK-GIRK cells used to create the action spectrum in A. **c** A light-intensity-response of the JellyOp cAMP response to a 5s light stimulus (show is mean ± SD). **d** JellyOp can be repetitively activated at a frequency that appears to be limited only by the speed at which GIRK currents recover in the dark (stimulation at 1min intervals). Green arrows are 5s illuminations at 500nm. Light intensities are indicated in panels (photons/cm^2/s). Source data are provided as a Source Data file.

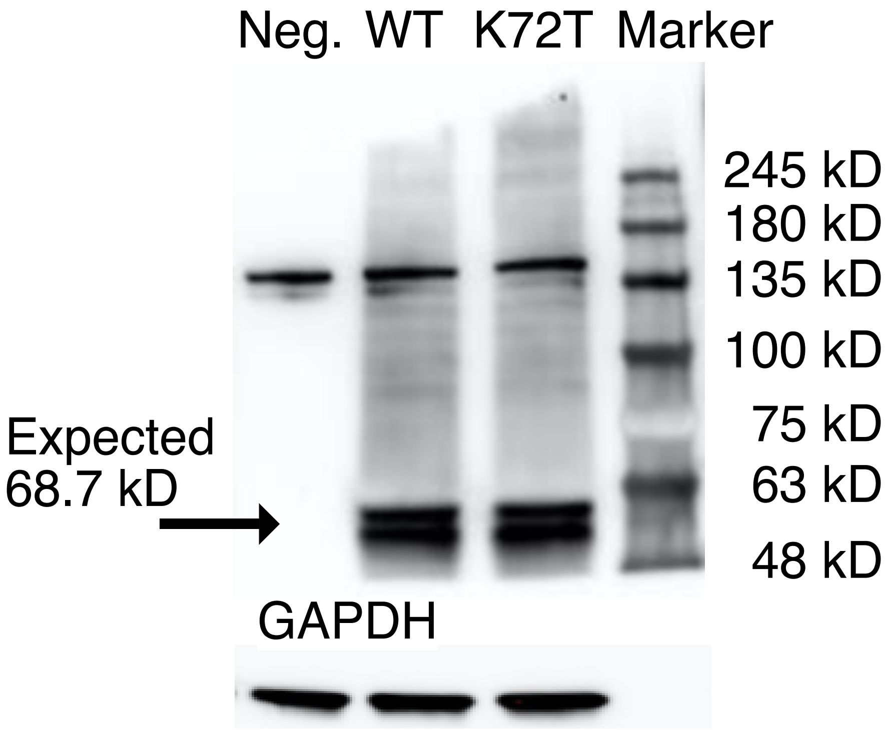

Supplementary Figure 2. **JellyOp does not covalently bind Gαs.** To exclude formation of an isopeptide bond between K72 of JellyOp and Q350 of Gαs, we transiently expressed JellyOp-mKate and JellyOp(K72T)-mKate in HEK293 cells for western blot analysis. Staining with an anti-mKate antibody showed that fusion proteins were of the expected size and not bound to Gαs. Protein double bands revealed differences in post-translational modification that was not investigated further. This experiment was repeated twice with similar results.

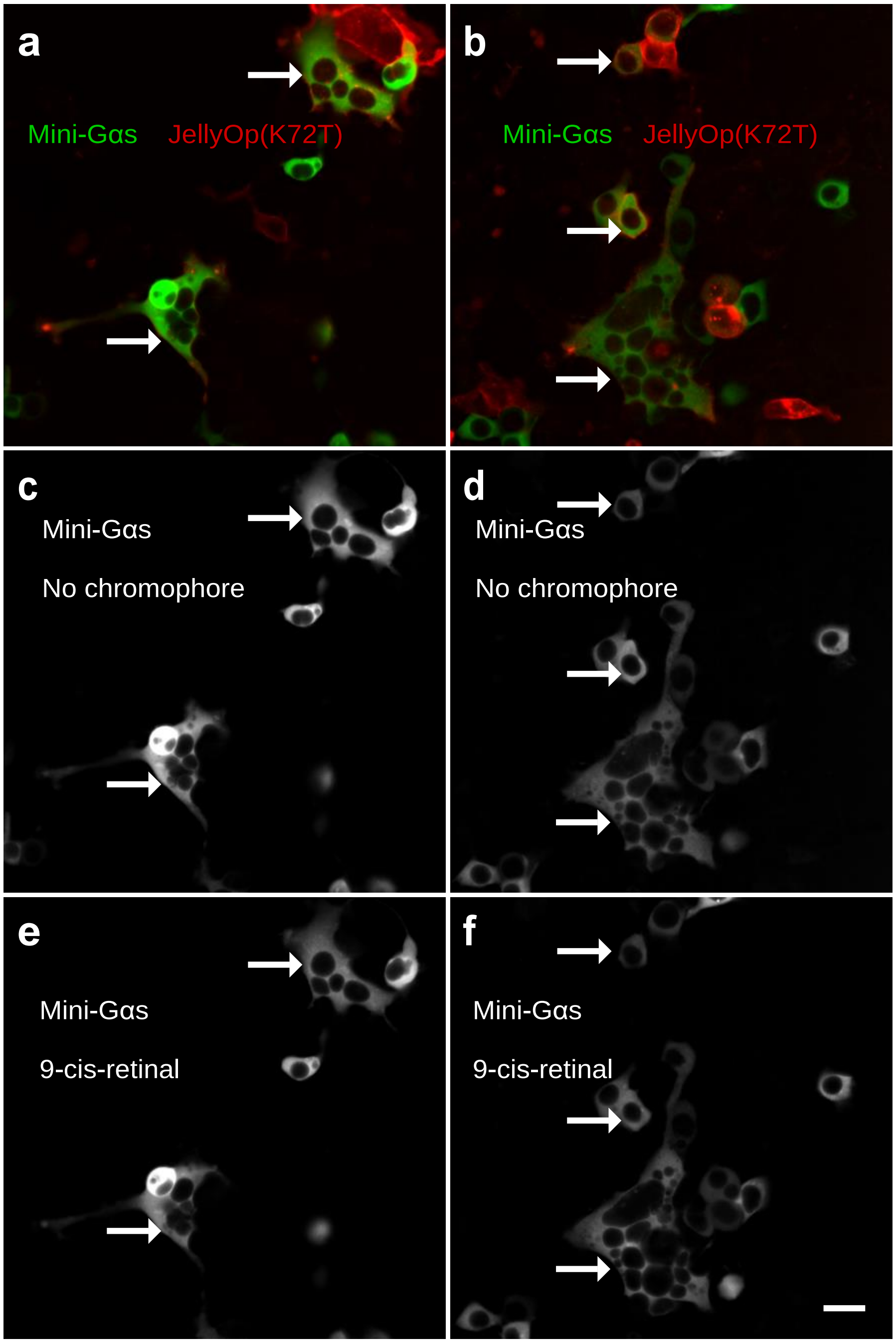

Supplementary Figure 3. **JellyOp(K72T) does not recruit NES-venus-MiniGαs to the cell membrane. a&b** HEK293 cells transiently transfected with NES-venus-MiniGαs (green) and JellyOp(K72T)-mScarlet (red), with some cells that express both plasmids (arrows). **c-f** The green channels from A and B are shown separately before (**c&d**) and after (**e&f**) adding 9-cis-retinal in the dark (1μM for 10min). NES-venus-MiniGαs was not recruited to the cell membrane after addition of 9-cis-retinal. Scale bar is 20μm. We repeated this experiment three times (independent recordings of cell populations) with similar results.

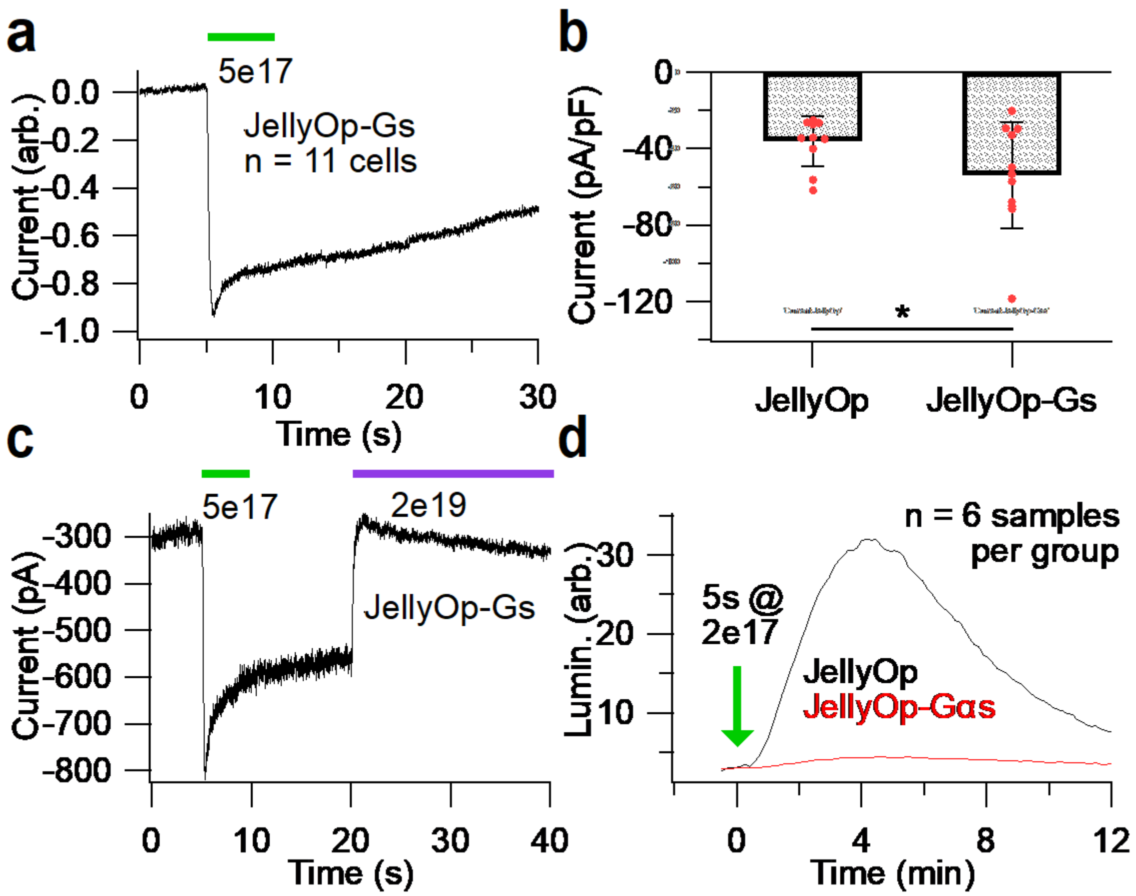

Supplementary Figure 4. **A JellyOp-Gαs fusion protein drives robust and selective Gβγ signalling. a** JellyOp-Gαs transiently transfected into HEK293-GIRK cells activates large and fast GIRK currents in response to green light. GIRK currents signalled by JellyOp-Gαs did not have a slow "sag" component (see Fig. 1) and was significantly larger than currents produced by WT JellyOp (**b**; p = 0.039; n = 10 JellyOp cells and 11 JellyOp-Gαs cells; bars show mean ± SD). **c** Similar to WT JellyOp, violet light rapidly inactivated GIRK currents driven by JellyOp-Gαs. **d** Despite strong coupling to GIRK (**a**-**c**), the same JellyOp-Gαs fusion protein did not modulate intracellular cAMP in response to light (error bands show mean ± SD). Light stimuli are shown as bars (green = 500nm; violet = 405nm) with intensities indicated on the figure panels (photons/cm^2/s). Source data are provided as a Source Data file.

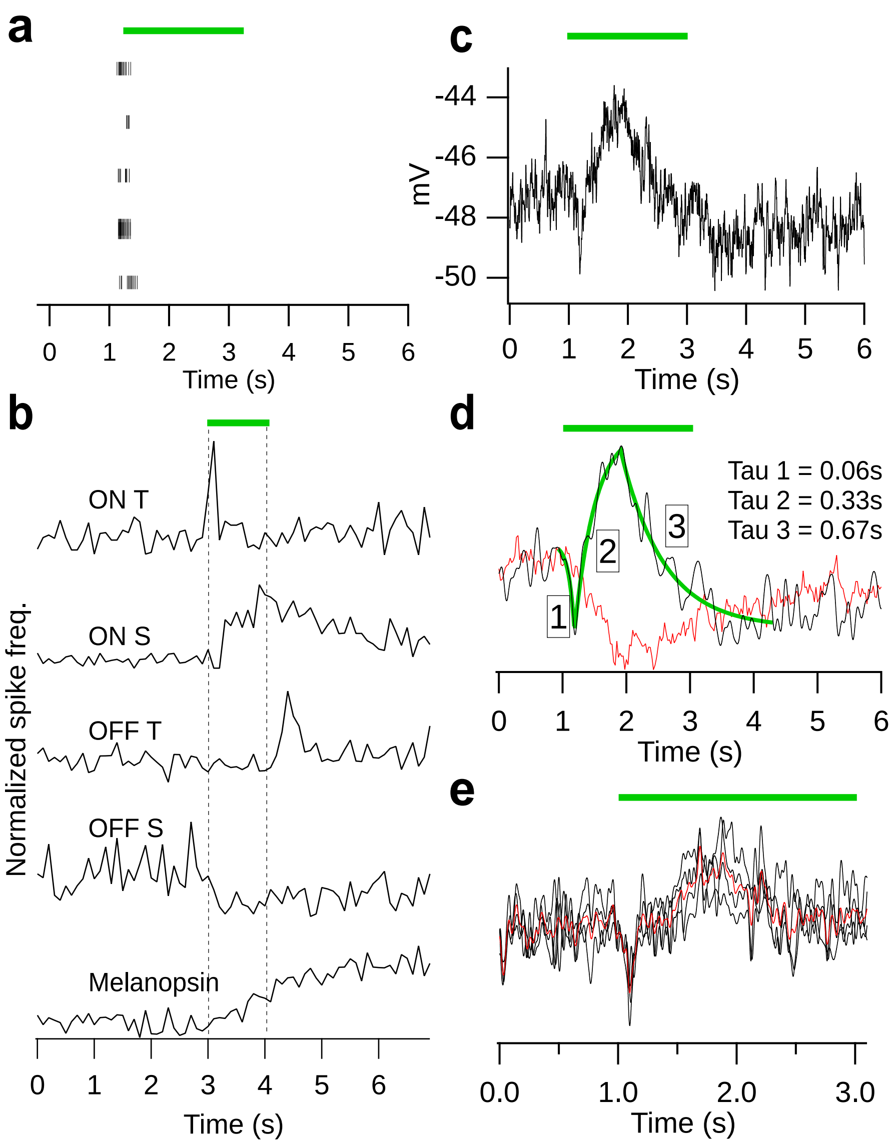

Supplementary Figure 5. **Electrical recordings from RGCs and rod-BCs of the JellyOp-treated rd1 retina. a** A raster plot shows reproducible spike activity of a single RGC in response to a 2s light flash presented at 10s intervals (green bar; 500nm). **b** Examples of RGCs with diverse spike responses to the same light stimulus. Traces show responses (top to bottom) of ON transient (ON T) ON sustained (ON S), OFF transient (OFF T), OFF sustained (OFF S) and melanopsin-like cells. Spike frequency histograms show the average of five traces recorded from the same cell. Bin width = 100ms. **c** An average voltage response recorded from rod-BCs transfected with JellyOp (single traces from n = 4 cells) show a fast transient depolarisation at the onset of a 2s light stimulus. **d** The rod-BC light response from **b** was filtered and fitted with single exponential functions to dissect various time constants of the response waveform (green). The average light response from rod-BCs transfected with Mela(CTmGluR6) is shown for direct comparison (red; n = 6). **e** Normalized and filtered single traces used to create average traces in **c&d**. Average (red) and standard deviation (shaded) is indicated.

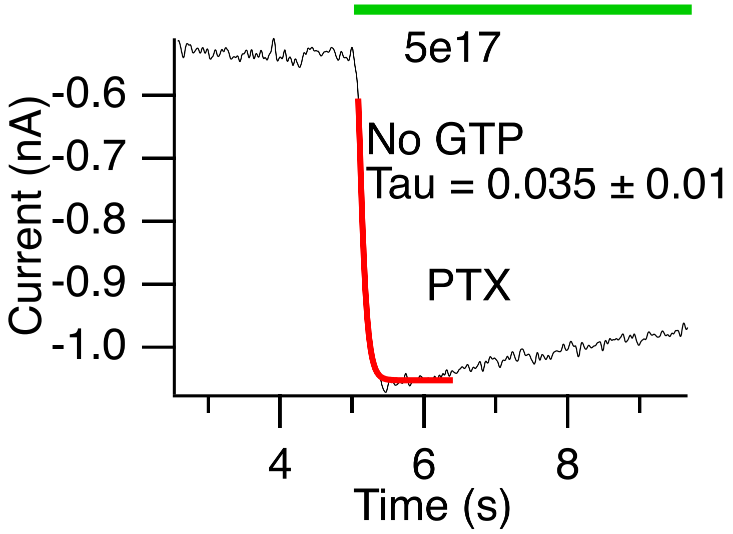

Supplementary Figure 6. **JellyOp still triggers fast GIRK currents in the absence of guanosine nucleotides.** A light-activated GIRK current from a HEK293-GIRK cell that was transiently transfected with JellyOp and path-clamped without GTP in the intracellular solution. To ensure wash-out of intracellular GTP, the cell was patch-clamped in a whole-cell configuration for 10min prior to the light stimulus. The onset of the light-triggered GIRK current remained characteristically fast. The light stimulus (500nm; 5×10^{17} photons/cm^2/s) is indicated above the trace.

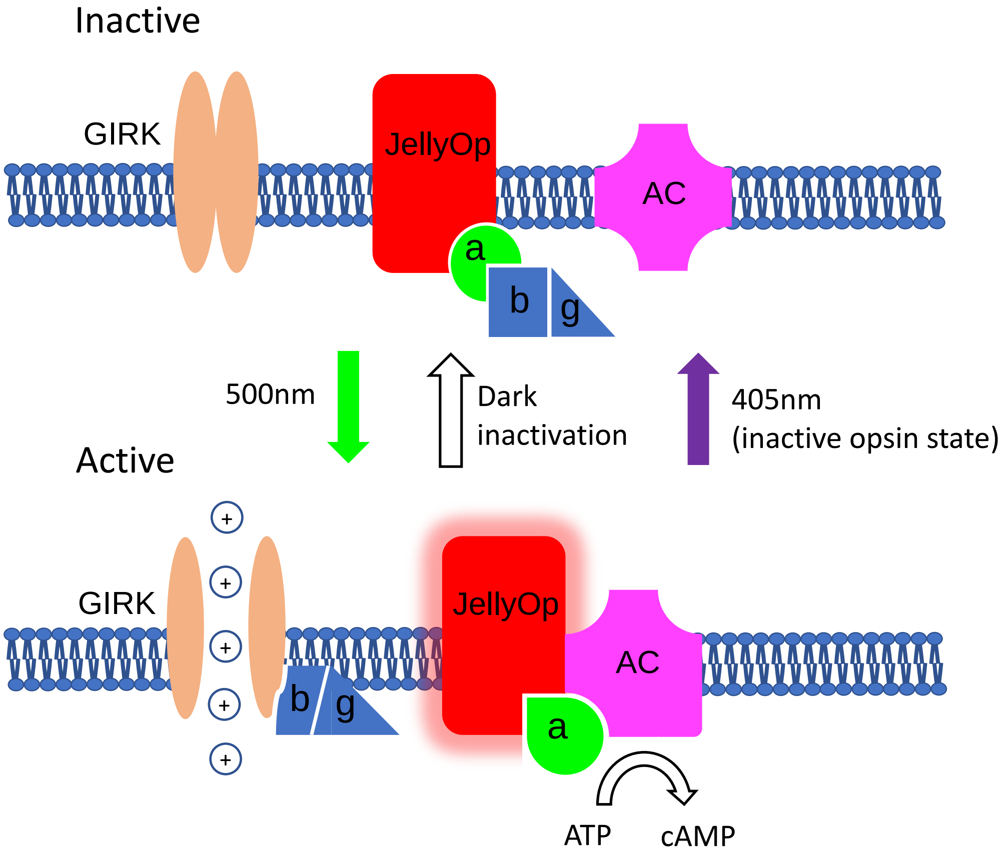

Supplementary Figure 7. **The unique G-protein coupling mode of JellyOp.** JellyOp constitutively binds a single Gαs partner throughout its inactive and signalling states. This creates a direct link between light detection and G-protein signalling. Since the active and inactive states of JellyOp respectively maintains the active and inactive states of Gαs, G-protein activity is independent of nucleotide exchange. This unconventional method of signalling infers that – in canonical GPCRs – nucleotide exchange might not be required for G-protein activation but only serve to keep G-proteins active long enough to assert their intended effect.

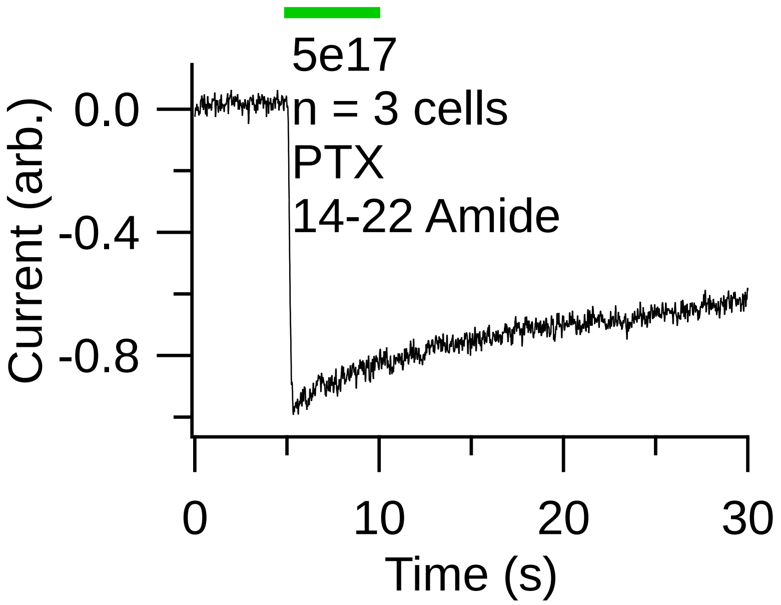

Supplementary Figure 8. **JellyOp-triggered GIRK currents are PKA independent.** Light-activated GIRK currents recorded from HEK293-GIRK cells that were transiently transfected with JellyOp and treated with Myr-PKI-14-22 Amide (10µM for 1h) before recording. Fast JellyOp-triggered GIRK currents persisted despite PKA block. The light stimulus (500nm; 5×10^{17} photons/cm^2/s) is indicated above the trace.

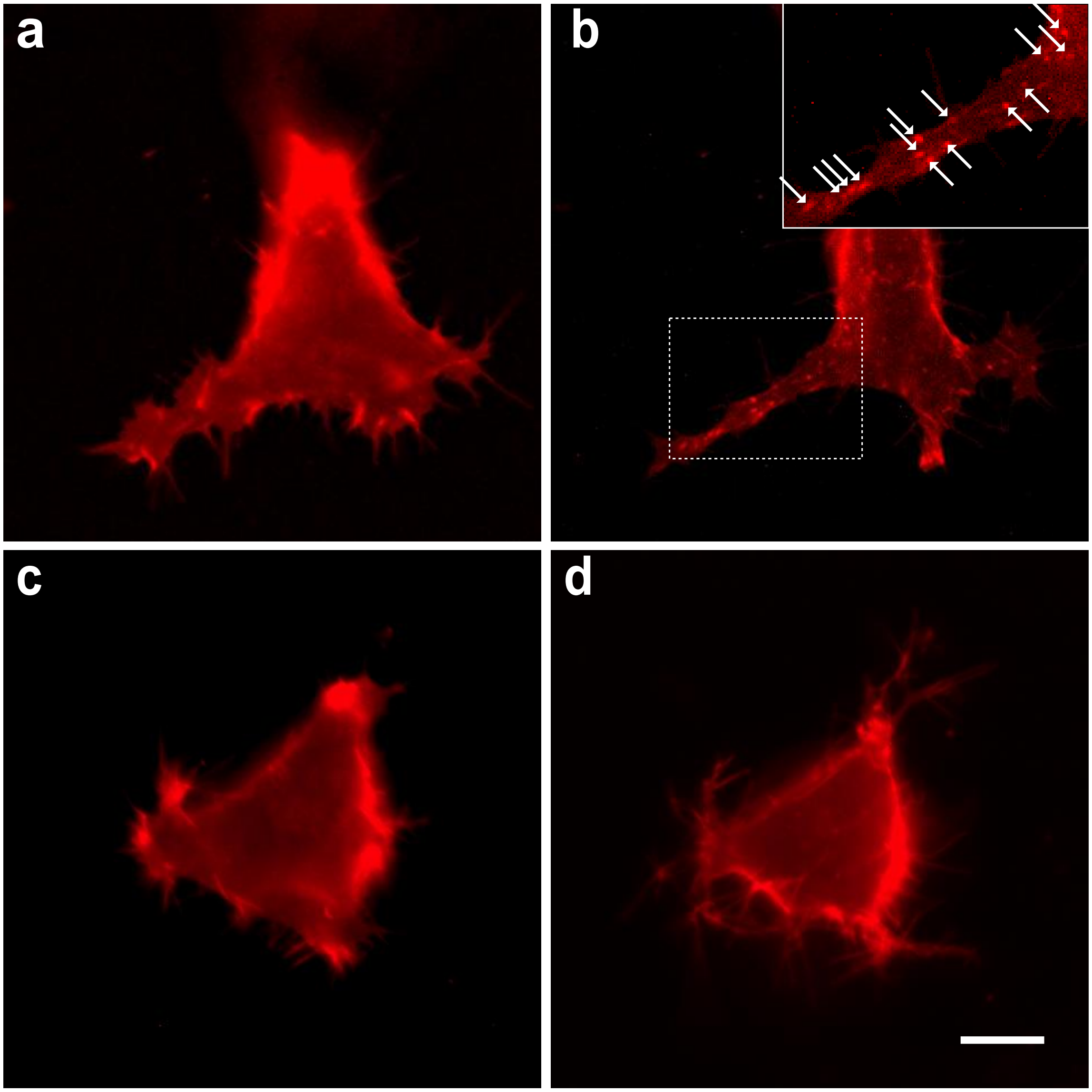

Supplementary Figure 9. **JellyOp does not undergo arrestin-mediated endocytosis after activation. a&b** Epi-fluorescence micrographs of the same HEK293 cell transfected with middle-wave cone opsin (OPN1MW-mKate) before (**a**) and 20min after (**b**) an intense light stimulation protocol (10×5s pulses at 1min intervals; 490nm; 1×10^{19} photons/cm^2/s). **b** (insert) 20min after light simulation the cell was filled with fluorescent vesicles indicative of opsin internalization (white arrows). **c&d** The same experiment from **a&b** performed on a cell expressing JellyOp-mKate before (**c**) and after (**d**) light stimulation. No noticeable internalization of opsin was observed (**d**). The scale bar in **d** is 10μm. Figures show examples of n = 4 cells tested per construct.
