# Supplementary material for: A visual opsin from jellyfish enables precise temporal control of G protein signalling
Source: Nat Commun. 2023 Apr 28;14:2450. doi: 10.1038/s41467-023-38231-z (PMC10147646; doi:10.1038/s41467-023-38231-z)
Supplement: Supplementary file 3 — Reporting Summary [file 41467_2023_38231_MOESM3_ESM.pdf]

nature portfolio

Corresponding author(s): Michiel van Wyk
Sonja Kleinlogel

Last updated by author(s): 27/3/23

Reporting Summary

Nature Portfolio wishes to improve the reproducibility of the work that we publish. This form provides structure for consistency and transparency in reporting. For further information on Nature Portfolio policies, see our Editorial Policies and the Editorial Policy Checklist.

Statistics

For all statistical analyses, confirm that the following items are present in the figure legend, table legend, main text, or Methods section.

n/a	Confirmed	
☐	☒	The exact sample size (*n*) for each experimental group/condition, given as a discrete number and unit of measurement
☐	☒	A statement on whether measurements were taken from distinct samples or whether the same sample was measured repeatedly
☐	☒	The statistical test(s) used AND whether they are one- or two-sided<br>*Only common tests should be described solely by name; describe more complex techniques in the Methods section.*
☐	☒	A description of all covariates tested
☐	☒	A description of any assumptions or corrections, such as tests of normality and adjustment for multiple comparisons
☐	☒	A full description of the statistical parameters including central tendency (e.g. means) or other basic estimates (e.g. regression coefficient) AND variation (e.g. standard deviation) or associated estimates of uncertainty (e.g. confidence intervals)
☐	☒	For null hypothesis testing, the test statistic (e.g. *F*, *t*, *r*) with confidence intervals, effect sizes, degrees of freedom and *P* value noted<br>*Give P values as exact values whenever suitable.*
☒	☐	For Bayesian analysis, information on the choice of priors and Markov chain Monte Carlo settings
☒	☐	For hierarchical and complex designs, identification of the appropriate level for tests and full reporting of outcomes
☒	☐	Estimates of effect sizes (e.g. Cohen's *d*, Pearson's *r*), indicating how they were calculated

Our web collection on statistics for biologists contains articles on many of the points above.

Software and code

Policy information about availability of computer code

Data collection	No custom software were used. We used HEKA PatchMaster 2.7, Striatech OptoDrum v 1.2.8 and Zeiss ZEN (black edition) for data collection.
Data analysis	No custom software were used. We used Igor Pro 7, Microsoft Excel v 16, R v 3.6.0 and ImageJ v 2.3 for data analysis.

For manuscripts utilizing custom algorithms or software that are central to the research but not yet described in published literature, software must be made available to editors and reviewers. We strongly encourage code deposition in a community repository (e.g. GitHub). See the Nature Portfolio guidelines for submitting code & software for further information.

Data

Policy information about availability of data

All manuscripts must include a data availability statement. This statement should provide the following information, where applicable:

- Accession codes, unique identifiers, or web links for publicly available datasets
- A description of any restrictions on data availability
- For clinical datasets or third party data, please ensure that the statement adheres to our policy

There are no restrictions to data availability. The source data used in graphs are available in a separate file. The DNA sequences for JellyOp and Mela(CT mGLuRs) are available in GenBank (accession codes AB435549.1 and MQ072285.1 respectively).

Human research participants

Policy information about studies involving human research participants and Sex and Gender in Research.

Reporting on sex and gender	N/A
Population characteristics	N/A
Recruitment	N/A
Ethics oversight	N/A

Note that full information on the approval of the study protocol must also be provided in the manuscript.

Field-specific reporting

Please select the one below that is the best fit for your research. If you are not sure, read the appropriate sections before making your selection.

☒ Life sciences ☐ Behavioural & social sciences ☐ Ecological, evolutionary & environmental sciences

For a reference copy of the document with all sections, see nature.com/documents/nr-reporting-summary-flat.pdf

Life sciences study design

All studies must disclose on these points even when the disclosure is negative.

Sample size	Sample sizes were mainly estimated form previous experience in experiments using similar experimental techniques (see for example doi: 10.1038/s42003-022-04016-1).
Data exclusions	No data were excluded from the analysis.
Replication	All experiments were repeated more than once and were reproducible. All data sets were presented with sample size as well as measure of variance.
Randomization	Animals were randomly allocated to treatment groups with no pre-selection criteria. We did not discriminate between male and female animals. In vitro comparisons of transfected cells were conducted in the same cell line and also in parallel on the same plate in plate reader experiments. Cells were randomly selected in patch-clamp experiments.
Blinding	No blinding was used. This was true for in vitro (plate reader and patch) experiments and for automated OMR tracking.

Reporting for specific materials, systems and methods

We require information from authors about some types of materials, experimental systems and methods used in many studies. Here, indicate whether each material, system or method listed is relevant to your study. If you are not sure if a list item applies to your research, read the appropriate section before selecting a response.

Materials & experimental systems

n/a	Involved in the study	
☐	☒	Antibodies
☐	☒	Eukaryotic cell lines
☒	☐	Palaeontology and archaeology
☐	☒	Animals and other organisms
☒	☐	Clinical data
☒	☐	Dual use research of concern

Methods

n/a	Involved in the study	
☒	☐	ChIP-seq
☒	☐	Flow cytometry
☒	☐	MRI-based neuroimaging

Antibodies

Antibodies used	anti-tRFP (Evrogen; AB234), donkey anti-rabbit Alexa488 (Invitrogen; AB_2535792), anti-GAPDH (Fitzgerald; 10R-G109A), anti-mouse HRP (Jackson Immuno Research; 115-035-146) and anti-rabbit HRP (Jackson Immuno Research; 111-035-144)
Validation	The specificity of AB234 for TurboFP635, as well as its use in immuno and western blot has been demonstrated (https://

Validation	evrogen.com/products/antibodies/AB-tRFP.shtml).<br>Human specificity of 10R-G109A, as well as its use in western blot has been demonstrated ((https://www.labome.com/product/Fitzgerald-Industries/10R-G109a.html)

Eukaryotic cell lines

Policy information about cell lines and Sex and Gender in Research

Cell line source(s)	The HEK293-GIRK cells were obtained from Dr. Olivia Masseck (Ruhr University Bochum).<br>HEK293 cells were obtained from Merck (85120602).<br>The HL-1 cell line was a gift from Nina Ulrich (University of Heidelberg).
Authentication	Robust GPCR triggered GIRK currents confirms the HEK-GIRK and HL-1 cell lines.
Mycoplasma contamination	Routine mycoplasma tests of all our cell lines confirmed no mycoplasma infection.
Commonly misidentified lines<br>(See ICLAC register)	We did not use commonly misidentified cell lines.

Animals and other research organisms

Policy information about studies involving animals; ARRIVE guidelines recommended for reporting animal research, and Sex and Gender in Research

Laboratory animals	Mice; C3H/HeOuJ (rd1), C57BL/6J (WT) and FVB/N Opto-mGluR6-IRES-Turbo635 (GM line with fluorescent OnBCs) strains.
Wild animals	No wild animals were used in this study.
Reporting on sex	The did not discriminate research animals based on sex.
Field-collected samples	No field-collected samples were used in this study.
Ethics oversight	Cantonal Veterinary Authority of Bern.

Note that full information on the approval of the study protocol must also be provided in the manuscript.
